# ^1^H, ^13^C, and ^15^N chemical shift assignments of monomorphic Aβ_1–40_ at 100 kHz MAS in a diamond rotor

**DOI:** 10.21203/rs.3.rs-11089530/v1

**Published:** 2026-10-07

**Authors:** Nicolas Matthey, Ravi Shankar Palani, Jiaming Liu, Salima Bahri, Robert Silvers, Brian Michael, Neil Gershenfeld, Sara Linse, Robert G. Griffin

**Affiliations:** a Dept. of Chemistry and Francis Bitter Magnet Laboratory, Massachusetts Institute of Technology Cambridge, MA 02139, USA; b Center for Bits and Atoms, Massachusetts Institute of Technology Cambridge, MA 02139 USA; c Dept. of Chemistry and Biochemistry, Florida State University, Tallahassee, FL 32306, USA; d Biochemistry and Structural Biology, Department of Chemistry, Lund University, Lund SE 22100, Sweden

**Keywords:** Amyloid fibrils, Magic-angle spinning NMR, ^1^H detection, Fast MAS, Diamond rotor

## Abstract

Amyloid-β (Aβ) fibrils are the principal component of the senile plaques that characterize Alzheimer’s disease, and fibrils formed from the Aβ_1–40_ alloform are strongly polymorphic, which has long complicated their structural characterization. We recently reported a reproducible, unseeded, monomorphic Aβ_1–40_ fibril grown at neutral pH from supersaturated monomer, together with its atomic-resolution structure and its ^13^C and ^15^N chemical shift assignments (PDB 12GB; BMRB 31300). Here we extend that reference to ^1^H. From a 3D ^1^H-detected hCCH (RFDR) experiment and a 2D hNH experiment recorded at 18.8 T and ω_r_/2π = 100 kHz on approximately 0.5 mg of hydrated fibrils packed in a home-built 0.7 mm single-crystal diamond rotor, we assign 123 ^1^H resonances, corresponding close to 90% of the distinct ^1^H sites of the rigid core (residues Q15→V40), together with sixteen ^1^H of the dynamic N-terminal segment. The ^1^H-detected data additionally yield six previously unassigned ^13^C and three previously unassigned ^15^N resonances, predominantly in aromatic side chains and in the mobile N-terminus, and nine ^1^H-^13^C and twenty ^1^H-^15^N inter-residue contacts that are consistent with the published structure. These assignments provide a residue-level reference for fingerprinting Aβ_1–40_ polymorphs and for mapping the binding of antibodies, chaperones and small molecules to this fibril.

## Biological context

Alzheimer’s disease (AD) is the most common cause of dementia, accounting for the majority of an estimated 55 million dementia cases worldwide (Nichols et al. 2022). A pathological hallmark of AD is the deposition of extracellular plaques composed primarily of the amyloid-β (Aβ) peptide (Chiti and Dobson 2006). Two alloforms predominate, Aβ_1–40_ and Aβ_1–42_ (Iwatsubo et al. 1994, Selkoe and Hardy 2016). Although Aβ_1–42_ aggregates more rapidly (Meisl et al. 2014) and is more neurotoxic (Klein et al. 1999), and has consequently received the most structural attention, Aβ_1–40_ is the more abundant species and is the major component of some plaques (Gravina et al. 1995). A molecular-level understanding of how both alloforms assemble, including their structures, aggregation mechanisms and dynamics, is essential for elucidating pathology and guiding therapeutic design. A central obstacle to this understanding is the structural polymorphism of the fibrils (Petkova et al. 2005, Bertini et al. 2011, Tycko 2015, Yang et al. 2022, Rodina et al. 2024). Aβ fibrils adopt distinct architectures depending on preparation conditions, and this heterogeneity is especially pronounced for Aβ_1–40_, where pH, ionic strength, peptide concentration, agitation, surfaces and the presence or absence of seeding can each select a different morph (Petkova et al. 2005, Tycko 2015). Cryo-electron microscopy (Cryo-EM) (Kollmer et al. 2019, Yang et al. 2022) and magic angle spinning (MAS) nuclear magnetic resonance (NMR) spectroscopy (Bertini et al. 2011, Lu et al. 2013, Colvin et al. 2015, Tycko 2015, Xiao et al. 2015, Colvin et al. 2016, Wälti et al. 2016, Silvers et al. 2017) studies have repeatedly shown the effect of small changes in the experimental conditions to the structure of the formed fibrils. Polymorphism not only complicates structure determination but also undermines reproducibility across laboratories, and addressing it requires well-defined model systems. For example, the GNNQQNY peptide, a polymorphic amyloid model has recently served as a testbed for such a solid-state NMR (ssNMR) structure determination approach (Mishra et al. 2025). A monomorphic Aβ_1–40_ reference prepared under physiological conditions is correspondingly valuable. In recent work we established such a reference: a reproducible, unseeded Aβ_1–40_ fibril formed at neutral pH from solutions of supersaturated monomer (Bahri et al. 2026), chosen to approximate the earliest molecular events, which are then propagated through secondary nucleation and elongation, in spontaneous assembly and to provide a defined baseline against which the effects of varied conditions, and of binding partners, can be measured.

Resolving such subtle structural differences requires a sensitive spectroscopic reporter. Among the NMR-active nuclei, ^1^H possesses a high sensitivity due to its high gyromagnetic ratio and is the most responsive to small changes in local structural environment due to the strong dependence of its chemical shift on hydrogen bonding and solvent exposure (Bahri et al. 2022, Le Marchand et al. 2022). This makes ^1^H shifts a discriminating probe of polymorphism and of binding-induced perturbations compared to ^13^C and ^15^N whose shifts can only modestly change even when the surface of the fibrils is engaged. The sensitivity has been exploited to map ligand and chaperone binding to Aβ fibrils. For instance, ^1^H-detected fast MAS NMR was recently used to study the interaction of the Bri2 BRICHOS domain with Aβ_1–42_ fibrils and to identify candidate aggregation hotspots on the fibril surface (Kumar et al. 2024). Similarly, MAS NMR of aducanumab bound to Aβ_1–42_ fibrils revealed that antibody engagement leaves the rigid core essentially unperturbed while markedly altering the dynamics of the surface-exposed N-terminal region, which is precisely the class of surface-localized change to which ^1^H shifts are the most sensitive (Palani et al. 2025). Complete ^1^H assignments are a prerequisite for such studies since without them, perturbations cannot be interpreted at the residue level.

^1^H detection in rigid solids has historically been limited by the strong ^1^H-^1^H dipolar couplings that broaden the spectra. MAS above ω_r_/2π =100 kHz, accessible with 0.7 mm rotors, attenuates these couplings sufficiently to yield resolved ^1^H resonances from fully protonated proteins. The experiments reported here were recorded in a custom designed 0.7 mm rotor machined from single-crystal high pressure, high temperature (HPHT) diamond. Diamond has a higher tensile strength than zirconia and is transparent to the millimeter-wave radiation used in dynamic nuclear polarization (DNP), so the assignments reported here are established on a platform that is also compatible with future DNP-enhanced acquisition (Schaffer et al. 2025).

Here we report near-complete ^1^H assignments, together with updated ^13^C and ^15^N assignments, for the monomorphic Aβ_1–40_ fibril. They were obtained from a 3D ^1^H-detected RFDR (hCCH) experiment and a 2D hNH experiment recorded at ω_r_/2π = 100 kHz MAS and 18.8 T on approximately 0.5 mg of hydrated fibrils packed in a laser machined diamond rotor. These assignments provide a residue-level reference for fingerprinting Aβ_1–40_ polymorphs and for mapping the binding of antibodies, chaperones, and other partners to this fibril.

## Methods and experiments

### Protein expression and purification

Aβ_1–40_ was expressed recombinantly in Escherichia coli BL21(DE3) pLysS star as previously reported (Bahri et al. 2026) from a construct carrying the autocleavable EDDIE mutant of N^pro^ as an N-terminal fusion partner (Achmüller et al. 2007); this construct yields peptide with the authentic Asp1 N-terminus. Uniform ^13^C,^15^N labelling was achieved by growing the cells in M9 minimal medium containing U-^13^C-glucose (2 g/l) and ^15^NH_4_Cl (1 g/l) as the sole carbon and nitrogen sources (LeMaster and Kushlan 1996, Castellani et al. 2002, Higman et al. 2009). Inclusion bodies containing the fusion protein were dissolved in 10 M urea, 10 mM Tris, 1 mM EDTA, 5 mM DTT, pH 8.3 and purified by anion-exchange chromatography in 4 M urea, 10 mM Tris, 1 mM EDTA, 5 mM DTT, pH 8.3 on two DEAE-Sepharose FF columns in tandem using a NaCl gradient from 150–350 mM in the same buffer, and the Aβ_1–40_ peptide was cleaved by dilution into urea-free Tris buffer (10 mM Tris, 1 mM EDTA, 5 mM DTT, pH 7.8). It was then purified to the monomeric form, after dialysis, by anion-exchange chromatography on Q-Sepharose big beads in batch mode, followed by two rounds of size-exclusion chromatography on a Superdex 75 26/600 column, with intervening lyophilization and dissolution in 6 M guanidinium chloride.

### Fibril formation and rotor packing

Monomeric Aβ_1–40_ from several repeats of the second size-exclusion step for each isotope combination, in 20 mM sodium phosphate, 0.2 mM EDTA, pH 7.4, was poured in a borosilicate glass bottle (Pyrex) and incubated under quiescent conditions at 37 °C, so that fibrils formed spontaneously from supersaturated solution. Fibril formation was confirmed by transmission electron microscopy (TEM). For the ^1^H-detected experiments reported here, hydrated fibril pellets containing approximately 0.5 mg of peptide were sedimented directly into a laser-machined 0.7 mm single-crystal diamond rotor in a Beckman Coulter ultracentrifuge at 55,000 rpm (SW60 rotor; corresponding to 310,680 average RCF) for 4 hour at 4 °C (Schaffer et al. 2025). The same fibril preparation supplied the ^13^C-detected data used here for comparison, for which approximately 20–30 mg of peptide were packed into 3.2 mm rotors (Bahri et al. 2026).

#### NMR spectroscopy

The ^13^C-detected spectra recorded in a 3.2 mm probe at ω_r_/2π = 20 kHz, and the ^13^C and ^15^N assignments derived from them that are used here for comparison, are described in (Bahri et al. 2026). All new spectra were recorded on a Bruker AVANCE III spectrometer operating at a static field of 18.8 T (800 MHz ^1^H Larmor frequency) using a triple-resonance (^1^H, ^13^C, ^15^N) 0.7 mm Bruker MAS probe. The sample was spun at ω_r_/2π = 100 kHz using a Bruker MAS 3 controller and the sample temperature was maintained at 277 K. The π/2 pulses were 0.775, 2, and 3 μs for ^1^H, ^13^C, and ^15^N respectively. ^13^C chemical shifts were referenced externally to adamantane (40.49 ppm for the downfield resonance) (Morcombe and Zilm 2003); ^1^H and ^15^N shifts were referenced indirectly using the IUPAC frequency ratios. The carrier frequencies were at 4.5 ppm for ^1^H, 100 ppm for ^13^C, and 120 ppm for ^15^N.

##### 3D ^1^H detected RFDR (hCCH)

The pulse sequence for this experiment (Golota et al. 2024) is displayed in Figure 1 with a wideband alternating-phase, low-power technique for zero-residual splitting (WALTZ) decoupling. Initial ^1^H to ^13^C cross polarization (CP) was achieved with 2 ms of contact time with a tangent ramp (± 10%) centered at $\omega_{1_{\text{H}}}/2\pi =182\;\text{kHz}$ with a constant ^13^C radio frequency (RF) at $\omega_{13_{\text{C}}}/2\pi =74\;\text{kHz}$. The back CP transfer from ^13^C to ^1^H was achieved with a 1.6ms contact time with a linear ^1^H ramp (± 5%) centered at $\omega_{1_{\text{H}}}/2\pi =158\;\text{kHz}$ and a constant ^13^C $\omega_{13_{\text{C}}}/2\pi =74\;\text{kHz}$. Low power WALTZ-16 (Shaka et al. 1983) decoupling using 10 kHz of RF was used during the t_1_ and t_2_ evolutions. The RFDR mixing evolution was achieved with a train of 4 μs π pulses for a mixing period of 1.92 ms. A multiple pulse solvent suppression sequence (MISSISSIPPI, 200 ms with an $\omega_{1_{\text{H}}}/2\pi =55\;\text{kHz}$ (Zhou and Rienstra 2008)) was used for water suppression.

##### 2D ^1^H detected hNH

The pulse sequence for this experiment is shown in Figure 2 (Le Marchand et al. 2022). Initial ^1^H to ^15^N CP was achieved with 1.5 ms of contact time with a tangent ramp (± 10%) centered at $\omega_{1_{\text{H}}}/2\pi =182\;\text{kHz}$ with a constant ^15^N radio frequency (RF) at $\omega_{15_{\text{N}}}/2\pi =38.94\;\text{kHz}$. The reverse transfer from ^15^N to ^1^H was achieved with a 1.4 ms contact time with a linear ^1^H ramp (± 5%) centered at $\omega_{1_{\text{H}}}/2\pi =158\;\text{kHz}$ and a constant ^15^N $\omega_{15_{\text{N}}}/2\pi =33.20\;\text{kHz}$. Low power WALTZ-16 (Shaka et al. 1983) decoupling using ω_1_/2π =10 kHz of RF was used during the t_1_ evolution. MISSISSIPPI (Zhou and Rienstra 2008) water suppression was employed for 200 ms with an $\omega_{1_{\text{H}}}/2\pi =55\;\text{kHz}$.

##### Data processing and analysis

Spectra were processed in TopSpin (Bruker) and assigned using CcpNmr Analysis (Skinner et al. 2016). Assignments were made from the ^13^C and ^15^N shifts deposited as BMRB entry 31300 (Bahri et al. 2026) as the starting point, as described in the following section.

### Extent of assignments and data deposition

The aim of this work is to add ^1^H detected experiments to the standard assignment workflow for amyloid fibrils. Both are built from conventional solid-state NMR blocks, cross polarization and RFDR, so they can either be run alongside a ^13^C-detected backbone walk assignment method or added to an already existing set of assignments. In either case, expanding existing assignments to include ^1^H shifts entail a modest investment in additional spectrometer time.

Sample preparation and the initial characterization of this fibril are described in (Bahri et al. 2026). ^13^C-^13^C RFDR and ^13^C INEPT-TOBSY spectra, recorded on both the uniformly ^13^C,^15^N-labeled sample and selectively labeled at H14, Q15, A21, V36, and G37, established that the preparation is monomorphic, and the ^13^C and ^15^N resonances were assigned with the standard suite of 3D NCACX, NCOCX and CONCA experiments. The atomic structure (PDB ID 12GB) was subsequently determined from ^13^C-^13^C distance restraints derived from proton assisted recoupling (PAR) and proton-driven spin diffusion (PDSD) and ^13^C-^15^N proton assisted insensitive nuclei (PAIN) experiments, together with TALOS-N torsion angles and small angle X-ray scattering. The ^13^C and ^15^N shifts were deposited as BMRB entry 31300. The same fibrils were then packed in a 0.7 mm diamond rotor and used to demonstrate that diamond rotors support high-resolution ^1^H-detected MAS NMR (Schaffer et al. 2025), but those ^1^H spectra were not assigned. The present work expands and provides an updated list of ^13^C and ^15^N assignments, adds the ^1^H assignments, and presents all three for this fibril.

The 3D hCCH RFDR spectrum is shown in Figure S3 and its ^13^C-^13^C projection is shown in Figure 3 and Figure 4. The ^13^C chemical shifts in this projection agree with those measured previously at ω_r_/2π = 20 kHz. Figure 5 shows residue by residue, the ^13^C chemical shift difference between the ^13^C-detected and ^1^H-detected experiments. All shifts agree to within 1 ppm, except Q15CB, L17C, D23CG and N27C. Most of these differences belong to the more labile side chains of the rigid-core residues, or to aromatic rings whose assignment is intrinsically ambiguous. The remainder are consistent with the higher sample temperature that accompanies ω_r_/2π=100 kHz MAS. The full list of resonances is given in Table S2.

Figure 6 shows the ^1^H-^13^C projection of the spectrum in Figure S3, taken along the second ^13^C dimension (F2), in which the directly bonded ^1^H-^13^C pairs appear with the highest intensity. Assignment proceeded from the strongest correlations where ^1^H directly bonded to already-assigned ^13^C were assigned first, and peaks closer to the noise floor, which correspond to longer-range correlations, were assigned subsequently. The ^1^H assignments in turn yielded four new ^13^C resonances absent in the ^13^C-detected data: D7CB, D7CG, H14CD2, H14CE1, F19CE2 and F20CG. H14CD2 and CE1 lie in the aromatic region, where ^13^C-detected assignment is intrinsically ambiguous. The complete list of assigned ^1^H resonances are given in Table S2.

The 2D hNH spectrum, shown in Figure 7, reproduces the ^15^N resonances reported previously and adds the ^1^H assignments of the backbone amides and of several side-chain NH groups. Differences between the ^15^N shifts measured here and those measured at ω_r_/2π=20 kHz are compiled in Figure 8. ^1^H detection allowed three new additional ^15^N resonances to be assigned, A2N, H14ND1, and H14NE2. The spectrum also shows cross peaks between backbone ^15^N and sidechain ^1^H of neighboring residues, which are discussed below,

Three of the newly assigned resonances lie in the N-terminal segment: the backbone amide of A2, the sidechain NH1 of R5, and D7CG. The N-terminus (D1→H14) is dynamic in this fibril and resonance from this section were largely absent from the CP-based spectra used for the structure determination; for example, A2, S8, G9 and K16 were detected only in the INEPT-TOBSY experiment (Bahri et al. 2026). Their appearance here, in CP-based experiments at ω_r_/2π = 100 kHz, indicates that part of this segment is accessible to dipolar-transfer methods under these conditions. The N-terminal region is the part of the fibril that engages antibodies and chaperones and the part that ^13^C-detected methods have described least well, so extending assignment into it is a practical benefit of ^1^H detection beyond the ^1^H shifts themselves.

In total, 123 ^1^H resonances were assigned from the two ^1^H-detected spectra which broken down by type represents 57.5% of backbone ^1^H^N^, 65% of ^1^H^α^ and 37.9% of side-chain ^1^H were assigned, alongside 79.5% of ^13^C and 73.1% of ^15^N. The vast majority of unassigned ^1^H cross peaks arise from the dynamic N-terminus of the protein as shown in the per-residue assignments in Figure 9. The remainder of the peaks are assigned, corresponding to close to 90% of the distinct ^1^H sites of the rigid core (residues 15–40), together with sixteen ^1^H from the dynamic N-terminal segment. The data also yielded inter-residues contacts, nine ^1^H-^13^C and twenty ^1^H-^15^N, which are shown on the published structure in Figure 10 and are in agreement with the structure. The full list of contacts is available in Table S4.

The ^1^H, ^13^C and ^15^N chemical shifts reported here have been deposited in the BMRB under accession 54013. Chemical shifts for the same fibril measured at ω_r_/2π = 20 kHz with ^13^C detection are available as BMRB entry 31300, which accompanies the structure determination (PDB 12GB).

## Conclusion

In summary, two ^1^H-detected experiments assign 83% of the rigid-core ^1^H sites, recovered ^13^C and ^15^N shifts that were absent from the ^13^C-detected data, and yielded inter-residues contacts consistent with the known structure. Added to a conventional assignment workflow, these experiments provide a more well-defined structure for a modest additional effort. The assignments reported here also establish a baseline against which other Aβ_1–40_ preparations, MAS rates, and sample temperatures can be compared.

## Supplementary Material

Supplementary Files

This is a list of supplementary files associated with this preprint. Click to download.

- 0SupplementaryInformationV16.docx

## Figures and Tables

**Figure 1:**
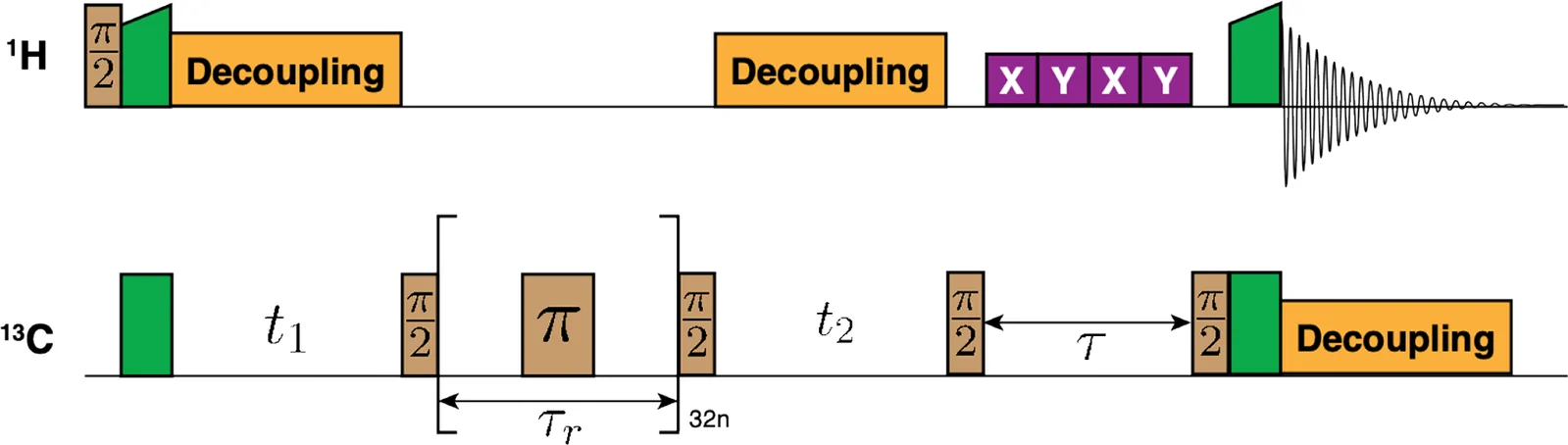
3D ^1^H detected hCCH-RFDR. The top line represents the ^1^H pulses and the bottom the ^13^C pulses. CP pulses are shown in green, decoupling in orange, π and π/2 pulses in brown and the water suppression in purple. τ_r_ is the rotor period. The RFDR block spans 32n rotor periods (integral n repetitions of the 32-step phase cycle) keeping the mixing time rotor-synchronized. τ is the water suppression period. The phase cycling is (in the order of pulses) ^1^H: π/2 = y,-y; ^1^H→^13^C CP = x; MISSISSIPPI = x, y, x, y; ^13^C→^1^H CP = [x]*4, [-x]*4; acquisition = x, -x, -x, x, -x, x, x, -x // ^13^C: ^1^H→^13^C CP = x; π/2 = y; RFDR π = x, y, x, y, y, x, y, x, y, -x, y, -x, -x, y, -x, y, -x, -y, -x, -y, -y, -x, -y, -x, -y, x, -y, x, x, -y, x, -y; π/2 = x, x, -x, -x; z-filter both π/2 = y; reverse CP = x.

**Figure 2:**
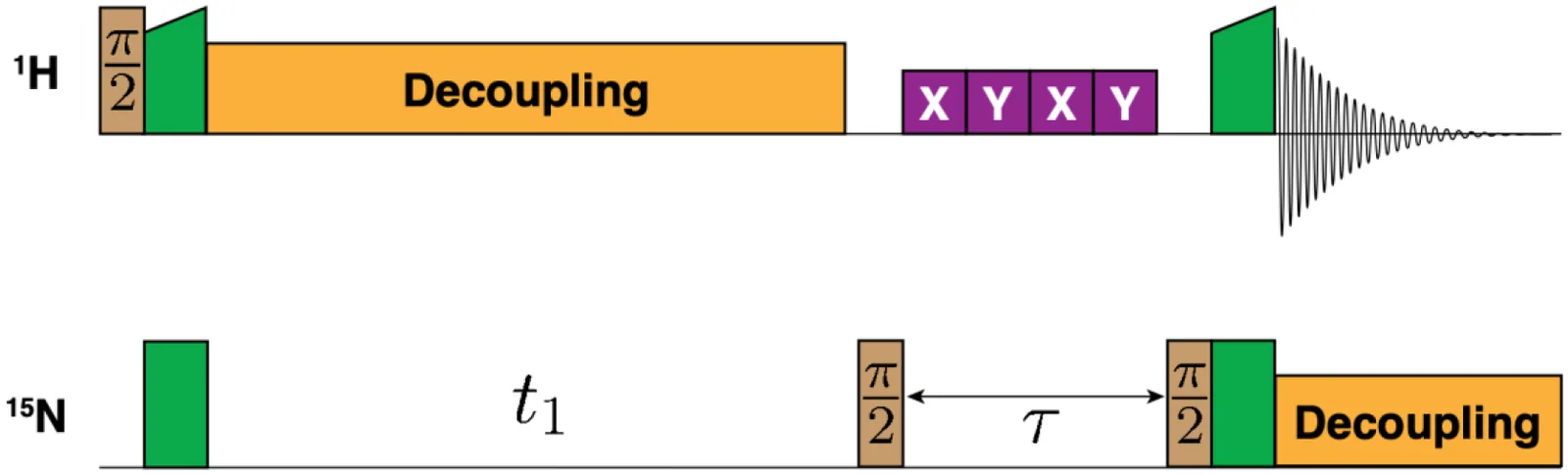
2D ^1^H-detected hNH. The first line represents the ^1^H pulses and the second line the ^15^N pulses. CP pulses are shown in green, decoupling pulses in orange, π and π/2 pulses in brown and the water suppression block in purple. τ represents the water suppression block. The phase cycling is (in the order of pulses) ^1^H: π/2 = y, -y; forward CP = x; MISSISSIPPI = x, y, x, y; backwards CP = y, y, y, y, -y, -y, -y, -y; acquisition = y, -y, -y, y, -y, y, y, -y // ^15^N: forward CP = y; first z-filter π/2 = x; second z-filter π/2 = x, x, -x, -x; backwards CP = y;

**Figure 3:**
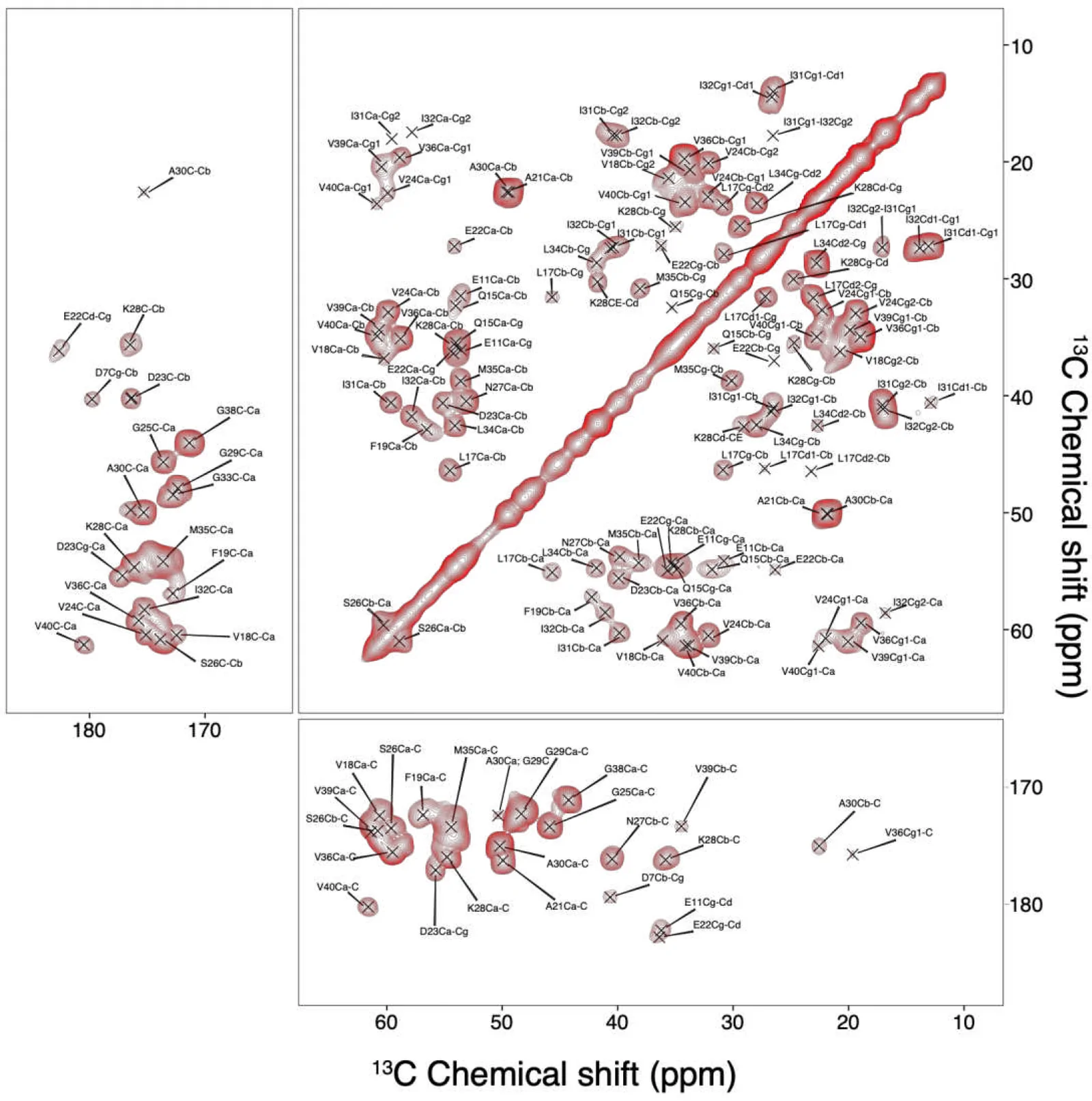
Projection onto the ^13^C-^13^C plane of the data acquired in Figure S3 showing the carbonyl and Cα.

**Figure 4:**
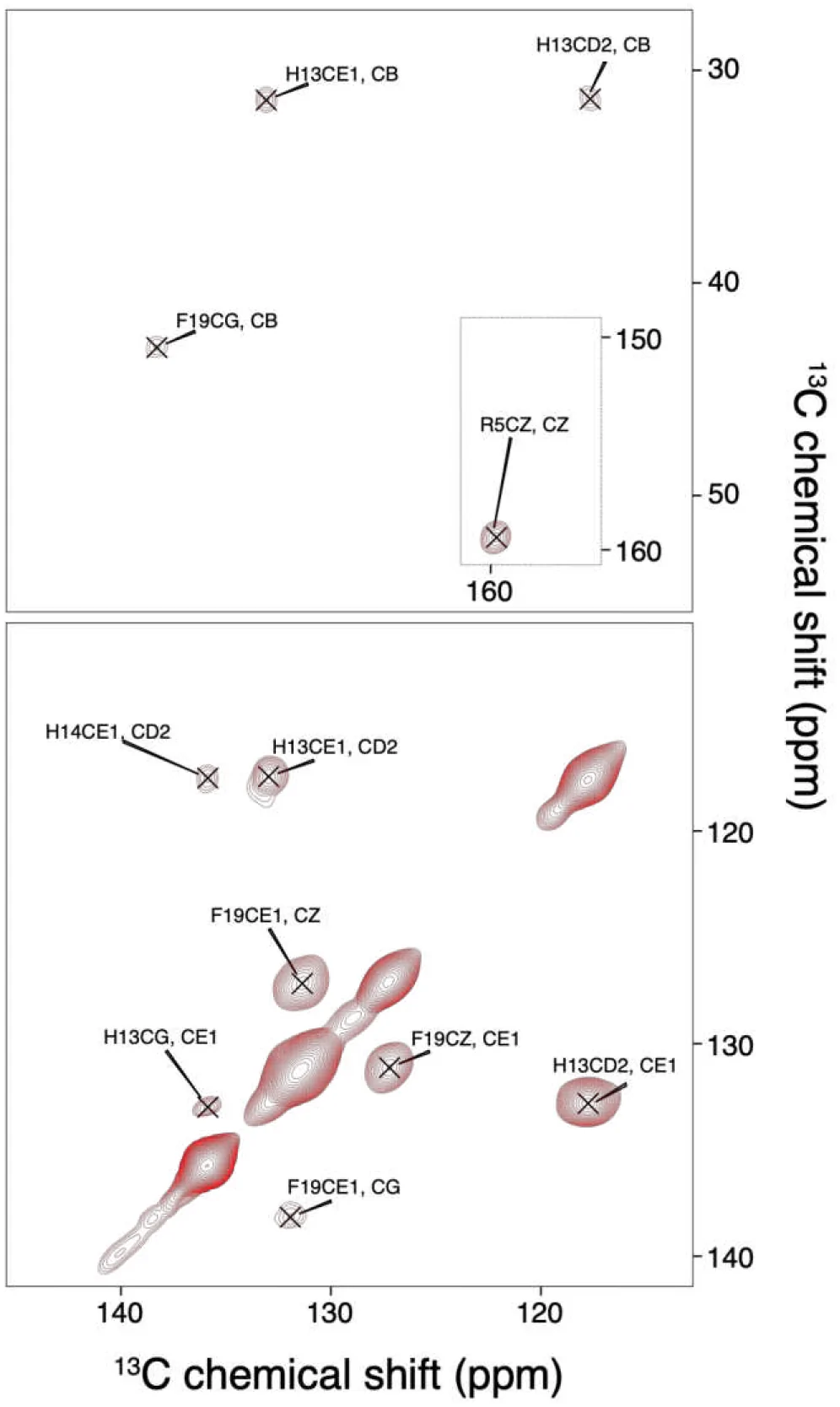
Projection of the ^13^C-^13^C plane of the data acquired in Figure S3 showing the aromatic region.

**Figure 5:**
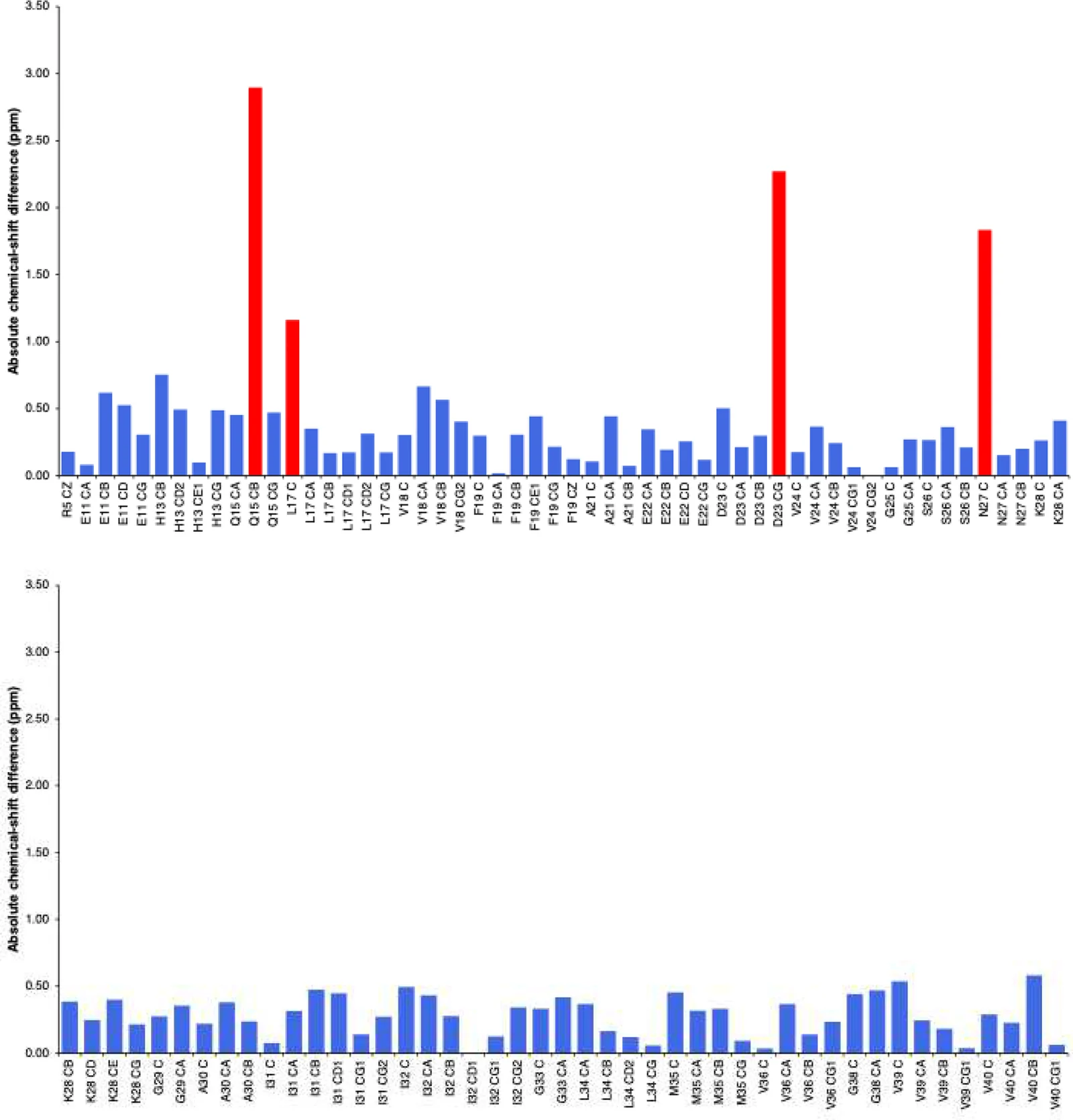
Difference between the ^13^C chemical shifts measured at ω_r_/2π = 20 kHz with ^13^C detection (Bahri et al. 2026) reported in table S1 and those measured here at ω_r_/2π = 100 kHz with ^1^H detection, shown for (top) residues R5-K28 and (bottom) K28-V40. Differences greater than 1 ppm are shown in red.

**Figure 6:**
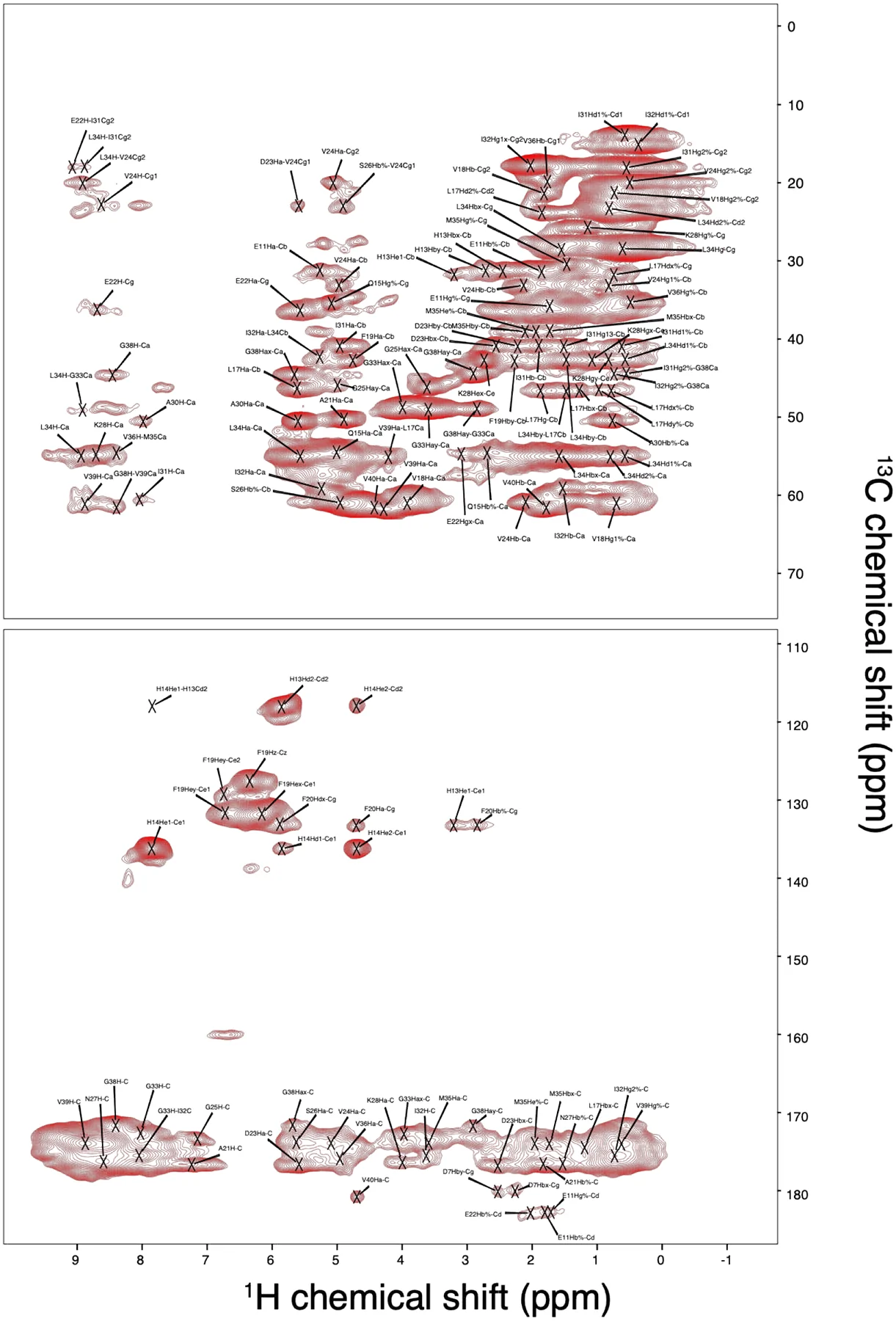
Projection onto the ^1^H-^13^C plane of the data acquired in Figure S3. This projection shows the 2^nd^ acquired ^13^C dimension and thus the transfer accomplished after the last ^13^C-^1^H cross polarization.

**Figure 7:**
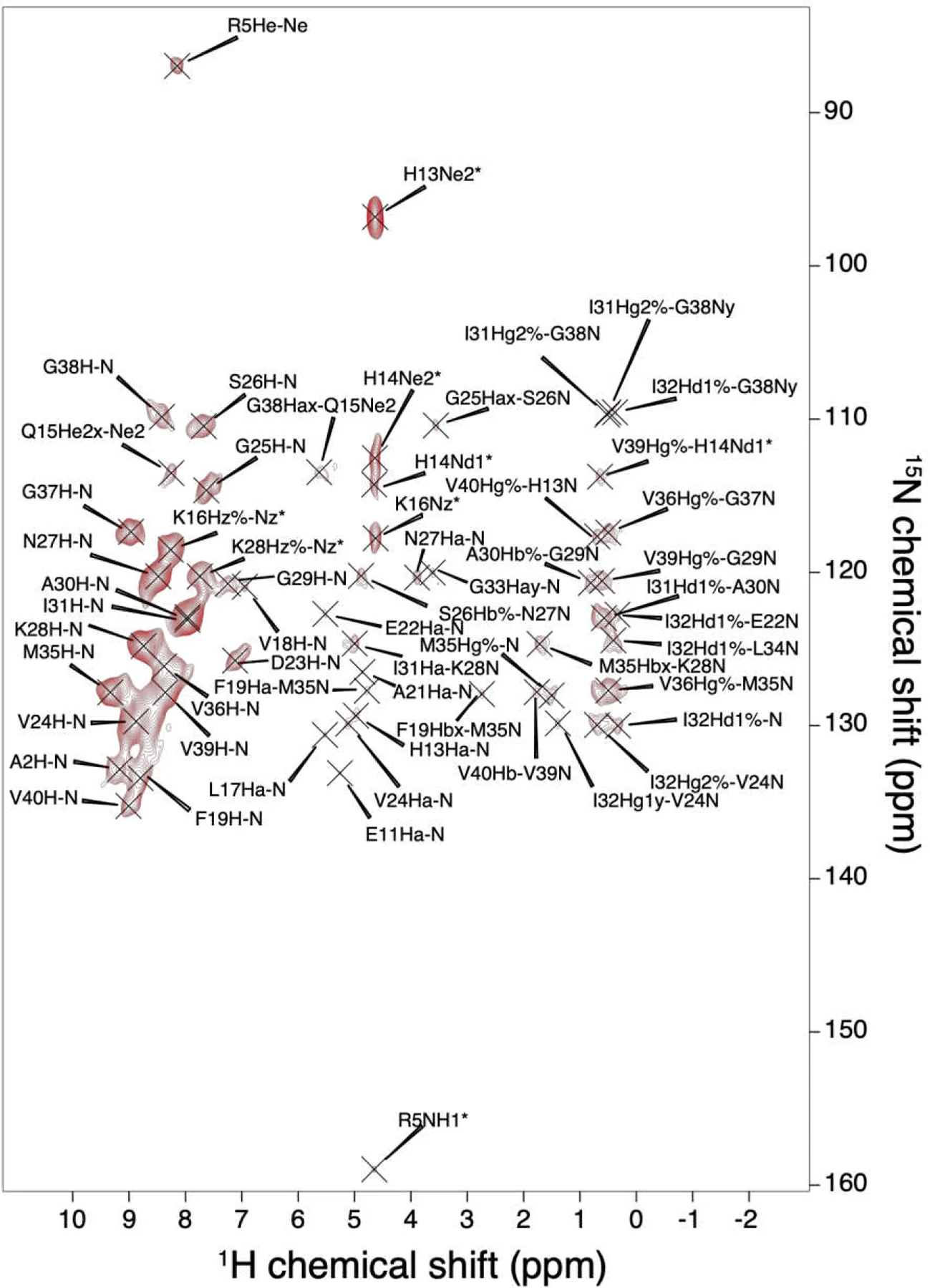
2D hNH spectrum of uniformly ^13^C-^15^N-labeled Aβ_1–40_. The spectrum was acquired at 18.8T, with ω_r_/2π = 100 kHz at 277 K. The folded peaks are denoted with an asterisk (*). The peaks not showing a ^1^H correlation are bound to the water from the environment.

**Figure 8:**
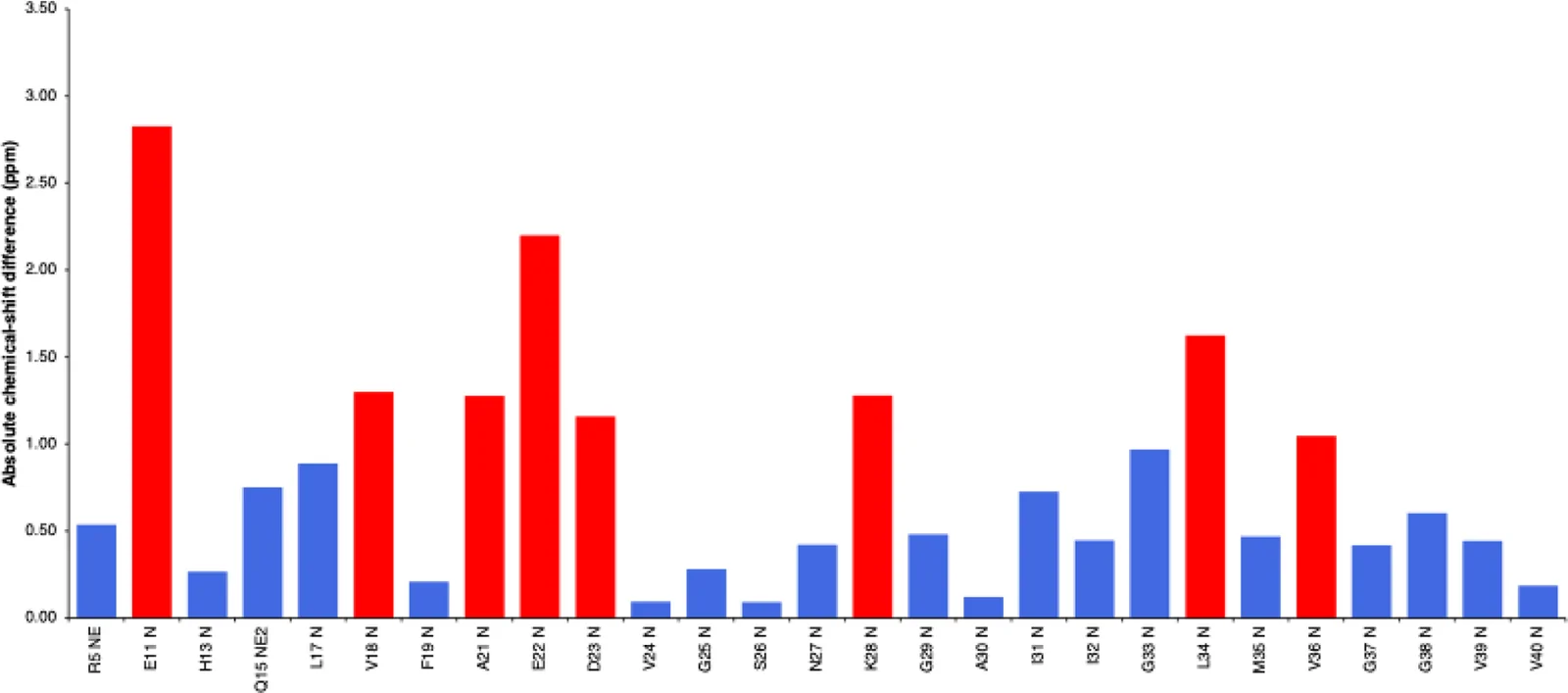
Bar chart showing the difference in chemical shift of the ^15^N peaks between the data acquired at ω_r_/2π =20 kHz (Bahri et al. 2026) reported in table S1 and the newly 100 kHz acquired data. Differences above 1 ppm are highlighted in red. The folded peaks were removed from the graph for readability (82 ppm difference).

**Figure 9:**
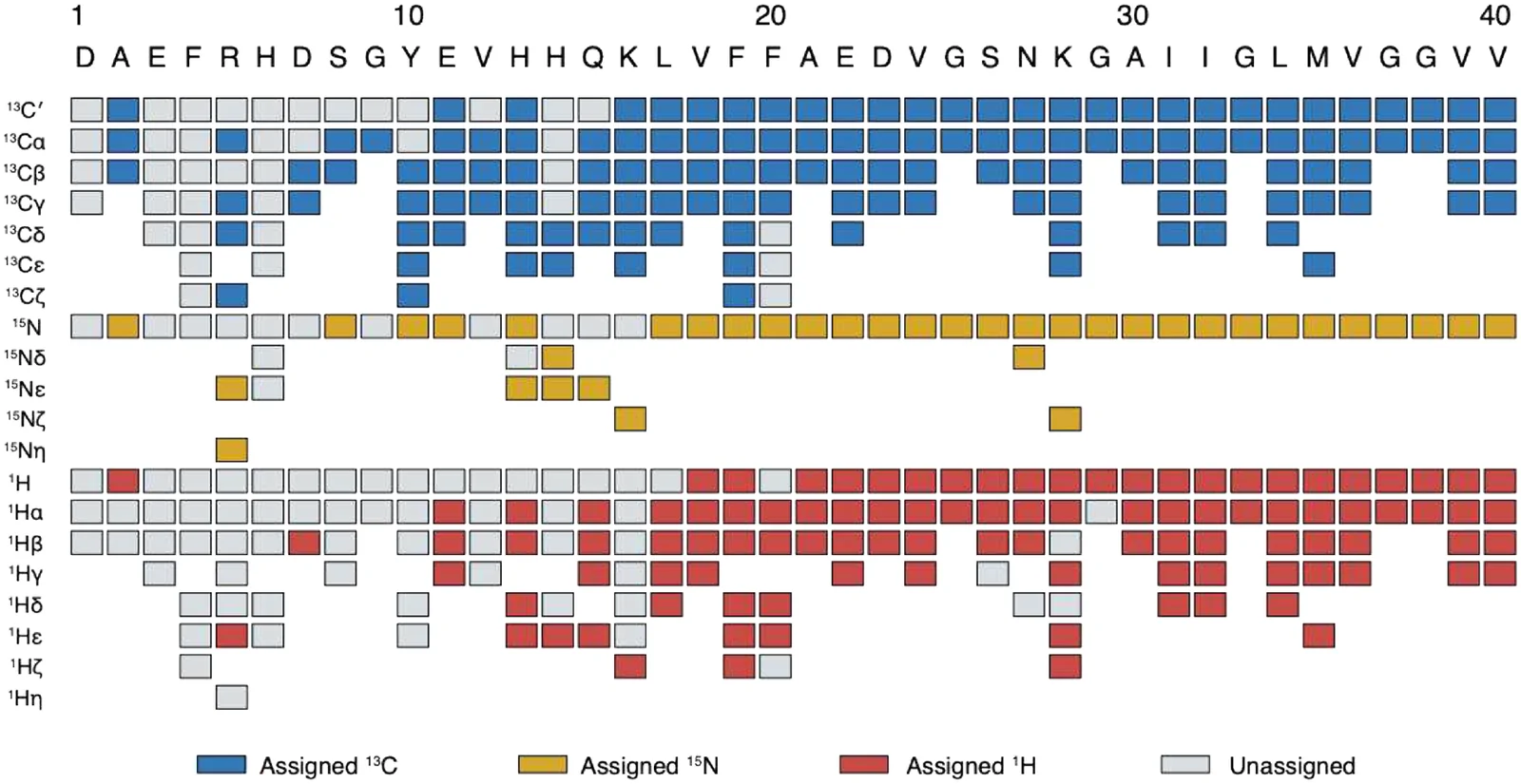
Schematic representation showing the assigned ^1^H, ^13^C and ^15^N nuclei of Aβ_1–40_.

**Figure 10:**
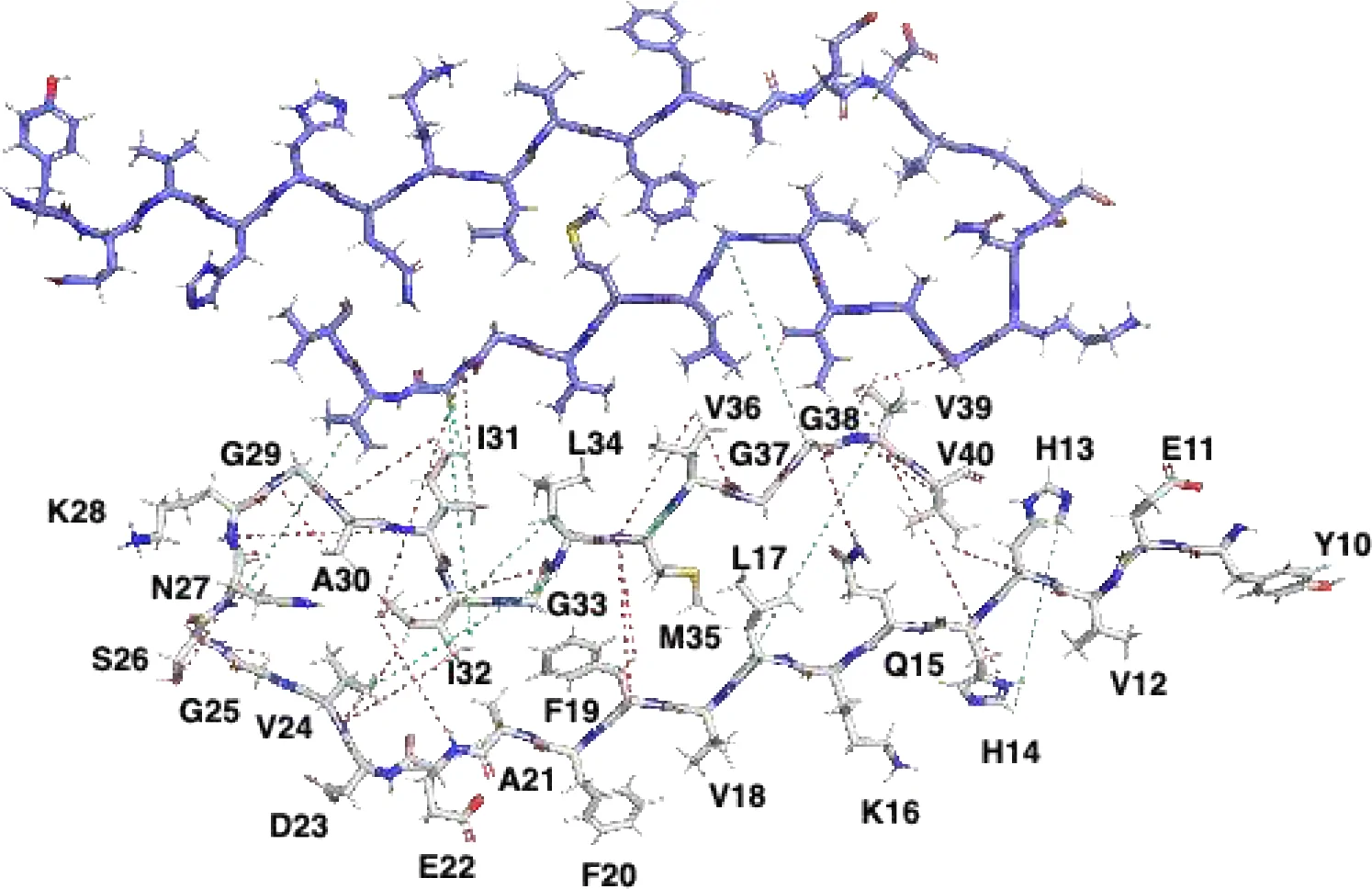
Lowest energy structures of Aβ_1–40_. Dashed lines show inter-residues contacts discovered by the ^1^H detected experiments. Green lines show ^1^H-^13^C contacts and red lines show ^1^H-^15^N contacts. Structure comes from PDB (12GB).

## Data Availability

^1^H, ^13^C and ^15^N new chemical shift assignments have been deposited in the BMRB under accession 54013. Previous ^13^C and ^15^N chemical shift assignments and atomic coordinates for the fibril structure are available in the BMRB under accession 31300 and the Protein Data Bank under accession 12GB, respectively.
